# Kinetic Model for the Direct Conversion of CO_2_/CO into Light Olefins over an In_2_O_3_–ZrO_2_/SAPO-34 Tandem Catalyst

**DOI:** 10.1021/acssuschemeng.3c06914

**Published:** 2024-01-18

**Authors:** Ander Portillo, Onintze Parra, Andres T. Aguayo, Javier Ereña, Javier Bilbao, Ainara Ateka

**Affiliations:** Department of Chemical Engineering, University of the Basque Country UPV/EHU, P.O. Box 644, Bilbao 48080, Spain

**Keywords:** kinetic model, deactivation, CO_2_ valorization, olefins, In_2_O_3_−ZrO_2_, SAPO-34, tandem catalyst

## Abstract

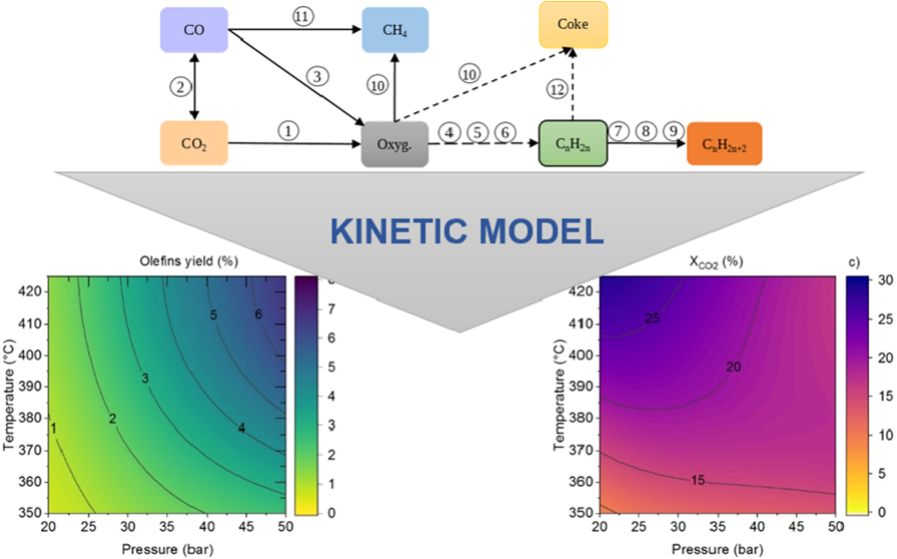

An original kinetic model is proposed for the direct production of light olefins by hydrogenation of CO_2_/CO (CO_*x*_) mixtures over an In_2_O_3_–ZrO_2_/SAPO-34 tandem catalyst, quantifying deactivation by coke. The reaction network comprises 12 individual reactions, and deactivation is quantified with expressions dependent on the concentration of methanol (as coke precursor) and H_2_O and H_2_ (as agents attenuating coke formation). The experimental results were obtained in a fixed-bed reactor under the following conditions: In_2_O_3_–ZrO_2_/SAPO-34 mass ratio, 0/1–1/0; 350–425 °C; 20–50 bar; H_2_/CO_*x*_ ratio, 1–3; CO_2_/CO_*x*_ ratio, 0–1; space time, 0–10 g_In2O3–ZrO2_ h mol_C_^–1^, 0–20 g_SAPO-34_ h mol_C_^–1^; time, up to 500 h; H_2_O and CH_3_OH in the feed, up to 5% vol. The utility of the model for further scale-up studies is demonstrated by its application in optimizing the process variables (temperature, pressure, and CO_2_/CO_*x*_ ratio). The model predicts an olefin yield higher than 7% (selectivity above 60%), a CO_*x*_ conversion of 12% and a CO_2_ conversion of 16% at 415 °C and 50 bar, for a CO_2_/CO_*x*_ = 0.5 in the feed. Additionally, an analysis of the effect of In_2_O_3_–ZrO_2_ and SAPO-34 loading in the configuration of the tandem catalyst is conducted, yielding 17% olefins and complete conversion of CO_2_ under full water removal conditions.

## Abstract 2

A kinetic model considering OX/ZEO catalyst deactivation is proposed for optimizing the direct formation of light olefins from CO/CO_2_ mixtures.

## Introduction

The processes for the direct production of fuels and chemicals by hydrogenation of CO_2_, such as the modified Fischer–Tropsch (MFT)^1^ and oxygenates (methanol/dimethyl ether (DME)) intermediated routes,^2^ are particularly attractive within the framework of Carbon Capture Utilization and Sequestration (CCUS) strategies.^3^ The route proceeding via oxygenates as intermediates requires OX/ZEO (metallic oxide/zeotype) tandem catalysts^4^ and enables the selective production of olefins,^5^ paraffins,^6^ aromatics,^7,8^ or C_5+_ hydrocarbons suitable for use as gasoline^9,10^ by selecting a zeotype with appropriate acidity and shape selectivity. A notable advantage of this process compared to the two-stage synthesis of methanol and subsequent conversion to hydrocarbons is that the *in situ* conversion of methanol/DME to hydrocarbons shifts the thermodynamic equilibrium of methanol synthesis.^11^

The processes of methanol/DME synthesis from CO_2_ and the conversion of oxygenates to hydrocarbons have reached considerable level and technological development.^12−17^ Similarly, the conversion of methanol to hydrocarbons has also progressed.^18−23^ However, the optimal conditions for the integrated process differ from those of the individual reaction stages. Moreover, the composition of the reaction medium is also different. Consequently, OX/ZEO tandem catalysts with different configurations, activities, selectivities, and stabilities have been designed for the specific reaction conditions of the integrated process.^24^

In previous studies, the excellent performance of the In_2_O_3_–ZrO_2_/SAPO-34 tandem catalyst for the selective production of light olefins was examined,^25^ based on the synergy between the CO_2_ adsorption capacity and hydrogenation activity of the oxygen vacancies on the In_2_O_3_–ZrO_2_ catalyst surface,^26^ and on the characteristic olefin selectivity of the CHA (Chabazite) structure of SAPO-34.^27^ Additionally, the ability of this catalyst to simultaneously convert CO_2_ and CO facilitates the cofeeding of syngas with CO_2_, which contributes to the sustainability process (when syngas is obtained from biomass or waste of the consumer society) and provides part of the required green H_2_. The negative synergistic effects of excessive concentration of H_2_O and methanol in the medium of the integrated process have also been studied. Particularly the features regarding the deactivation of the tandem catalyst, which affects the reaction conditions,^28^ the configuration of the tandem catalyst (OX/ZEO ratio), and the arrangement in the reactor, which must be appropriate to mitigate deactivation.^29^

In this paper, an original kinetic model has been established, which is essential for the scale-up of the process. Note that previous studies on the kinetic modeling of the direct synthesis of hydrocarbons from CO_2_ over tandem catalysts are scarce. Ghosh et al.^30^ proposed a kinetic model for the conversion of CO_2_ to a broad range of hydrocarbons (C_2_–C_9+_, including aromatics) over an In_2_O_3_/HZSM-5 tandem catalyst. The reaction network consists of 21 elementary steps (simplified to 11), considering the reactions catalyzed by the In_2_O_3_ and the HZSM-5 catalysts. The rate kinetic equations follow a Langmuir–Hinshelwood (LH) type, including terms that quantify the extent of reaction limitation by reactant adsorption on the active sites of each individual catalyst. To simplify the calculation of the kinetic parameters, previously obtained for the reactions involved in methanol synthesis catalyzed by In_2_O_3_^31^ were used and those for the reactions activated by HZSM-5 zeolite were calculated. Cordero–Lanzac et al.^32^ established a kinetic model for the direct formation of C_2_–C_4_ paraffins from CO_2_/CO over a PdZn/ZrO_2_ + SAPO-34 tandem catalyst. The reaction network consists of seven reaction steps, considering the reactions activated by the PdZn/ZrO_2_ catalyst (methanol synthesis from CO_2_, reverse Water Gas Shift (rWGS), methanation, and formation of each C_2_–C_4_ paraffin from methanol). In this model, the expressions of the reaction rates follow LH-type equations and quantify the limitations of the reaction extent due to reactant and H_2_O adsorption. It is noteworthy that these kinetic models in the literature^30,32^ do not consider catalyst deactivation, nor the cofeeding of CO, which are original aspects considered in this manuscript. The applicability of the model to tandem catalysts with different loadings of In_2_O_3_–ZrO_2_ and SAPO-34 is also an original contribution. The versatility of the kinetic model is demonstrated by applying it to analyze the effect of reaction variables, such as pressure, temperature, and space time of each individual catalyst, while also considering the cofeeding of CO along with CO_2_.

## Experimental Section

Tandem catalysts were obtained by physically mixing the In_2_O_3_–ZrO_2_ and SAPO-34 (ACS Material) catalysts with particle sizes in the range of 125–250 and 300–400 μm, respectively. The preparation of In_2_O_3_–ZrO_2_ was previously described in detail elsewhere by Portillo et al.^26^ Briefly, a coprecipitation using In(NO_3_)_3_ (Sigma-Aldrich), Zr(NO_3_)_4_ (Panreac), and NH_4_CO_3_ (Panreac) was carried out. The fresh and deactivated catalysts were extensively characterized in previous studies,^26,27^ and their properties are presented in Section S1 (Table S1).

The kinetic runs were conducted using a PID Eng. & Tech. Microactivity Reference reactor setup, as detailed in Portillo et al.,^25^ employing an isothermal fixed-bed reactor under a wide range of operating conditions: In_2_O_3_–ZrO_2_/SAPO-34 mass ratio, 0/1–1/0; 350–425 °C; 20–50 bar; H_2_/CO_*x*_ molar ratio in the feed, 1–3; CO_2_/CO_*x*_ molar ratio in the feed, 0–1; space time (defined as the ratio between the mass of each individual catalyst and the molar flow of CO_2_/CO at the reactor inlet), 0–10 g_In2O3–ZrO2_ h mol_C_^–1^, 0–20 g_SAPO-34_ h mol_C_^–1^; time on stream, up to 500 h; H_2_O and CH_3_OH in the feed, up to 5% vol. For the analysis of the composition of the feed and product streams, a Varian CP-4900 (Agilent) microchromatograph was used, as described by Portillo et al.^25^

In Section S2, Table S2 specifies the conditions used for each experimental run considered for the modeling. Note that several runs were repeated to ascertain the reproducibility of the experiment. As an example of the reproducibility of the runs, the results of three reactions carried out at certain conditions are displayed in Figure S1. Moreover, the absence of mass transfer constraints for the catalysts was ascertained in Section S3, both theoretically, according to the compliance with the Weisz–Prater criteria (eq S1) for the internal diffusion, and also experimentally, as illustrated in Figures S2 and S3, for internal and external diffusion, respectively.

The modeling was carried out fitting the molar fractions of the components, and the simulation results were quantified by calculating olefin yields, *Y*_*i*_ (eq S2), CO_2_ conversion, *X*_CO2_ (eq S3), and CO_*x*_ conversion, *X*_CO*x*_ (eq S4) based on the molar flows of the reactants and products in C content units as defined in Section S4.

## Results and Discussion

### Reaction Network

**Figure 1:**
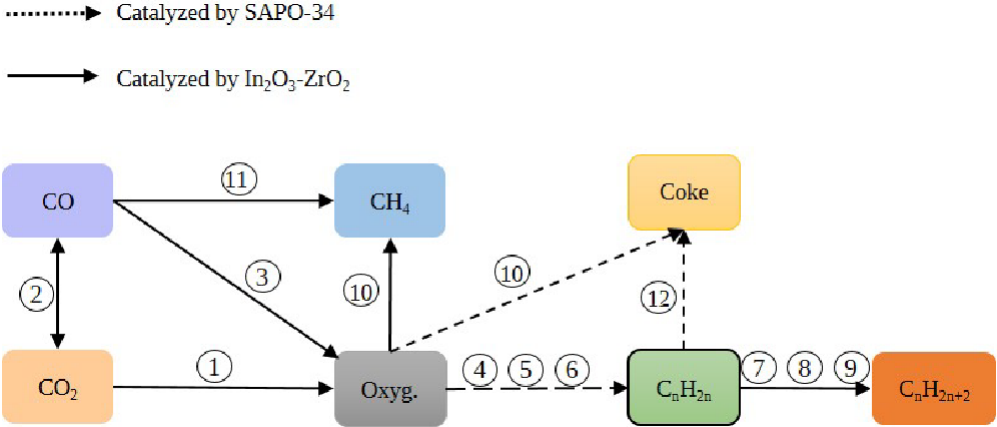
Proposed reaction network. The numbers correspond to the individual reactions in Table 1.

The reaction network, as illustrated in Figure 1, gathers the reactions outlined in Table 1. The In_2_O_3_–ZrO_2_ catalyzes the methanol synthesis (steps 1, 3; eqs 1 and 3), reverse Water Gas Shift (rWGS) (eq 2), hydrogenation of olefins to paraffins (steps 7–9; eqs 7–9), and methanation (step 11, eq 11) reactions. On the other hand, SAPO-34 catalyzes the formation of olefins from methanol/DME, as described in steps 4–6 (eqs 4–6), with methanol as the reactant, considering DME as an intermediate given its more reactive nature.^33^ The decomposition (thermal cracking) of oxygenates (step 10; eq 10), contributes to the formation of CH_4_. The reaction network also incorporates the pathways for coke formation, including the decomposition of oxygenates (step 10, eq 10) and the dehydrocyclization of olefins, leading to the formation of aromatics as intermediates, which subsequently condense into polyaromatic species.^34^

**Table 1:** Individual Reactions Involved in the Reaction Network of Figure 1 and Corresponding Kinetic Equations

reaction	kinetic equation
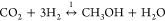 1	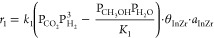 13
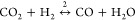 2	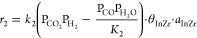 14
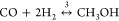 3	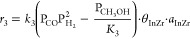 15
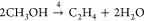 4	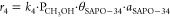 16
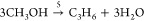 5	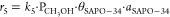 17
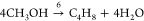 6	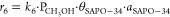 18
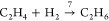 7	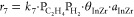 19
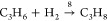 8	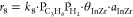 20
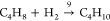 9	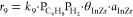 21
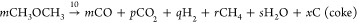 10	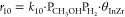 22
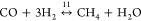 11	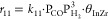 23
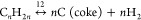 12	

### Kinetic Equations

The expressions for the kinetics reaction rates are described in Table 1 (eqs 13–23) along with the corresponding individual reaction. The terms θ_InZr_ and θ_SAPO-34_ consider that the extent of the individual reactions in Table 1 catalyzed by In_2_O_3_–ZrO_2_ and SAPO-34 catalysts, respectively, is limited by the competitive adsorption of different components in the reaction medium on the active sites. This leads to the formulation of a Langmuir–Hinshelwood (LH)-type kinetic model. The best fit of the kinetic model was determined with the following expressions (eqs 24 and 25). Cordero–Lanzac et al.^32^ justify the expressions in eqs 24 and 25 by emphasizing the reaction mechanisms and the role of CO_2_ and H_2_ adsorption as rate-limiting steps in methanol synthesis, as well as the role of H_2_O adsorption in limiting SAPO-34 activity in the conversion of oxygenates to olefins.
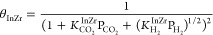
24
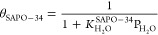
25

The deactivation of each catalyst by coke in the tandem catalyst is considered in eqs 13–21 and 23 with two activity terms, *a*_InZr_ and *a*_SAPO-34_, corresponding to the In_2_O_3_–ZrO_2_ and SAPO-34 catalysts, respectively. These terms are defined as reaction rate ratios, as defined by Levenspiel et al.,^35^ and are calculated as described by Cordero–Lanzac et al.^36^ to take into account the *past history* of the catalyst. The deactivation kinetic expressions are dependent on the concentration of methanol, which is the main precursor of coke:
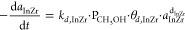
26
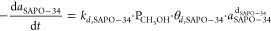
27

In these equations, the terms θ_*d*,IZr_ and θ_*d*,SAPO-34_ quantify the limitation of the extent of coke formation reactions on the In_2_O_3_–ZrO_2_ and SAPO-34 catalysts, respectively, due to the presence of H_2_O and H_2_, as described by the following expressions:
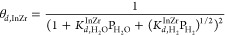
28
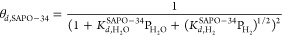
29

Eqs 26 and 27, along with their complementary counterparts, eqs 28 and 29, align with the results of coke deposition determined in a previous study through TPO analysis of the two catalysts employed under different conditions.^28^ Notable findings from these results include: (i) higher coke deposition in SAPO-34; (ii) the influence of methanol concentration favoring coke formation, while H_2_O mitigates this formation; and (iii) the converging trend of coke content evolution over time on stream toward a limiting value.

Moreover, it must be mentioned that the role of H_2_O^28^,^37−40^ and H_2_^29^,^41−43^ as mitigating agents of coke deposition in the conversion of methanol/DME over acid catalysts is well established. Presumably, the role of H_2_O in attenuating the condensation of coke intermediates, and of H_2_ in facilitating their hydrogenation, will be similar for the coke deposited on the In_2_O_3_–ZrO_2_ catalyst, thereby mitigating its deactivation.

In the calculations, temperature-dependent equilibrium constants for methanol synthesis reactions (steps 1 and 3 in Figure 1) and rWGS (step 2) were used, as proposed by Ateka et al.^12^ These expressions were calculated from standard values of formation enthalpies and entropies for the components of each individual reaction.^44^

The mathematical approach used for analyzing the kinetic data in this study has been described in other catalytic processes,^36,45,46^ and briefly in Section S5.

### Fitting of the Model and Apparent Kinetic Parameters

To evaluate the strong agreement between the calculated molar fractions of the components in the outlet stream of the reactor and the corresponding experimental data, parity plots are presented in Figure S4 (Section S6). The quality of the fit between the calculated and experimental results falls within the typical ranges found in the literature for kinetic models of other catalytic processes with complex reaction schemes that consider catalyst deactivation.^36,47−49^

The values of the apparent kinetic parameters resulting in the best fit (with a minimal error objective function, eq S6, corresponding to a confidence degree of 95%) are presented in Table 2. The model simplification study (based on the significance test explained in Section S5), verified that the fit did not improve using different apparent activation energies for the conversion of CO_2_ and CO into methanol (steps 1 and 3, respectively, in Figure 1). This lack of improvement may be attributed to their relationship through the rWGS reaction (step 2). Similarly, there was no significant enhancement in the model fit when considering different apparent activation energies in the individual formation of olefins (steps 4–6) and paraffins (steps 7–9), and in the formation of CH_4_ through decomposition of methanol (step 10) and oxygenates (step 11). Consequently, the kinetic constants for these steps with the same apparent activation energies are expressed in Table 2 relative to the highest value within the group of reactions sharing the same activation energy. Additionally, constants with the same activation energy are specified at the bottom of the Table 2.

**Table 2:** Parameters of the Kinetic Model (Units at the End)^a^

Apparent kinetic parameters^b^		
	k_j_*	*E*
*k*_2_	4.0 × 10^–2^ ± 7.0 × 10^–4^	8.1 × 10^0^ ± 1.2 × 10^0^
*k*_3_	2.3 × 10^–5^ ± 5.2 × 10^–7^	1.8 × 10^0^ ± 4.0 × 10^–2^ (same for *k*_1_)
*k*_5_	3.6 × 10^0^ ± 4.9 × 10^–4^	7.2 × 10^0^ ± 1.9 × 10^0^ (same for *k*_4_ and *k*_6_)
*k*_8_	7.4 × 10^–2^ ± 1.5 × 10^–3^	7.3 × 10^–2^ ± 6.1 × 10^–4^ (same for *k*_7_ and *k*_9_)
*k*_10_	1.7 × 10^–2^ ± 7.2 × 10^–4^	9.2 × 10^1^ ± 1.5 × 10^1^

Apparent adsorption parameters		
	K*_ads_	Δ*H*
*K*_CO_2__^InZr^	5.1 × 10^0^ ± 5.9 × 10^–1^	–5.1 × 10^0^ ± 9.6 × 10^–1^
*K*_H_2__^InZr^	7.6 × 10^–3^ ± 4.3 × 10^–4^	–1.7 × 10^2^ ± 3.8 × 10^0^
*K*_H_2_O_^SAPO–34^	3.1 × 10^–4^ ± 7.4 × 10^–5^	–9.4 × 10^–1^ ± 2.9 × 10^–1^

Apparent deactivation parameters		
	k*_d,j_	*E*_*d*_
*k*_*d*,InZr_	5.7 × 10^–3^ ± 1.7 × 10^–3^	2.0 × 10^0^ ± 6.4 × 10^–2^
*k*_*d*,SAPO-34_	5.7 × 10^3^ ± 2.5 × 10^2^	1.4 × 10^–2^ ± 5.0 × 10^–3^

Apparent adsorption parameters for deactivation		
*K*_*d*,H_2_O_^InZr^	4.1 × 10^–2^ ± 4.2 × 10^–4^	–2.7 × 10^2^ ± 8.2 × 10^1^
*K*_*d*,H_2__^InZr^	4.2 × 10^–3^ ± 1.0 × 10^–3^	–4.0 × 10^1^ ± 9.8 10^0^
*K*_*d*,H_2_O_^SAPO–34^	6.8 × 10^–3^ ± 1.3 × 10^–3^	–4.2 × 10^0^ ± 9.1 × 10^–2^
*K*_*d*,H_2__^SAPO–34^	2.8 × 10^–4^ ± 1.1 × 10^–4^	–1.3 × 10^1^ ± 2.8 × 10^0^

Deactivation order	
d_InZr_	1.2 ± 1.7 × 10^–2^
d_SAPO-34_	2.6 ± 1.5 × 10^–2^

a Units: *k*_1,11_: mol_C_ g^–1^ h^–1^ bar^–4^; *k*_2,8_: mol_C_ g^–1^ h^–1^ bar^–2^; *k*_3,7,9,10_: mol_C_ g^–1^ h^–1^ bar^–3^; *k*_4–6,*d*_: mol_C_ g^–1^ h^–1^ bar^–1^; *E*_a_, Δ*H*, *E*_d_: kJ mol^–1^; K: bar^–1^.

b Note: *k*_*1*_ = 0.01 *k*_*3*_; *k*_*4*_ = 0.36 *k*_*5*_; *k*_*6*_ = 0.30 *k*_*5*_; *k*_*7*_ = 0.36 *k*_*8*_; *k*_*9*_ = 0.11 *k*_*8*_*; k*_*11*_ = 10^–5^*k*_*10*_

Further comparing the values in Table 2, the apparent constant related to the formation of oxygenates from CO_2_ is even 100 times lower than that corresponding to the formation from CO (k*_3_ = 2.3 × 10^–5^ mol_C_ g^–1^ bar^–3^). This result confirms that methanol formation reaction will be the key conditioning factor in the extent of the process and that the rWGS reaction plays a crucial role in converting the CO_2_/CO mixture into oxygenates, as established in the literature.^11^

Comparing the apparent activation energy values of each reaction, the highest value (92.5 kJ mol^–1^) corresponds to CH_4_ and coke formation from oxygenates (*E*_10_), which is in line with the thermal nature of this reaction, that requires temperature control to minimize its extent. The low apparent activation energy values in deactivation kinetics can be justified in catalytic processes involving coke deactivation, where the coke formation/elimination mechanism is complex, and reaction temperature has different effects in reactions of oligomerization, cyclization, dehydrogenation, cracking, etc., justify the apparent activation energy.^50,51^

The remarkable difference between the deactivation constant of SAPO-34 at the reference temperature (5.7 × 10^3^ mol_C_ g^–1^ bar^–1^) and the negligible value for the In_2_O_3_–ZrO_2_ catalyst (5.7 × 10^–3^ mol_C_ g^–1^ bar^–1^) is consistent with previously reported coke deposition results in both catalysts.^28^ Moreover, comparing the effect of H_2_O adsorption on olefin formation reactions: (= 3.1 × 10^–4^ bar^–1^) and deactivation of SAPO-34 (= 6.8 × 10^–3^ bar^–1^), it is observed that the presence of H_2_O offers more advantages (referred to deactivation attenuation) than disadvantages (attenuation of olefin formation). The deactivation kinetics order for the In_2_O_3_–ZrO_2_ catalyst (1.2) indicates an almost linear dependence of the deactivation rate on the remnant activity in eq 26. However, the deactivation order for SAPO–34 in eq 27 is 2.6, indicating a deactivation mechanism with rapid pore blockage by coke deposition, which is typical for this catalyst in the conversion of oxygenates to olefins,^52−54^ but maintaining a remnant activity. This tendency is in good agreement with the evolution of coke deposition over time on stream.^28^

Figure S5 displays a covariance matrix relating the kinetic parameters at reference temperature. These information ascertained there is not significant codependence between steps in the reaction mechanism apart from those already considered (correlation between the olefin formation steps 4–6; the steps of paraffin formation 7–9, and the two steps of methanol formation, thus, the formation from CO_2_ (step 1) and the formation from CO (step 3)).

**Figure 2:**
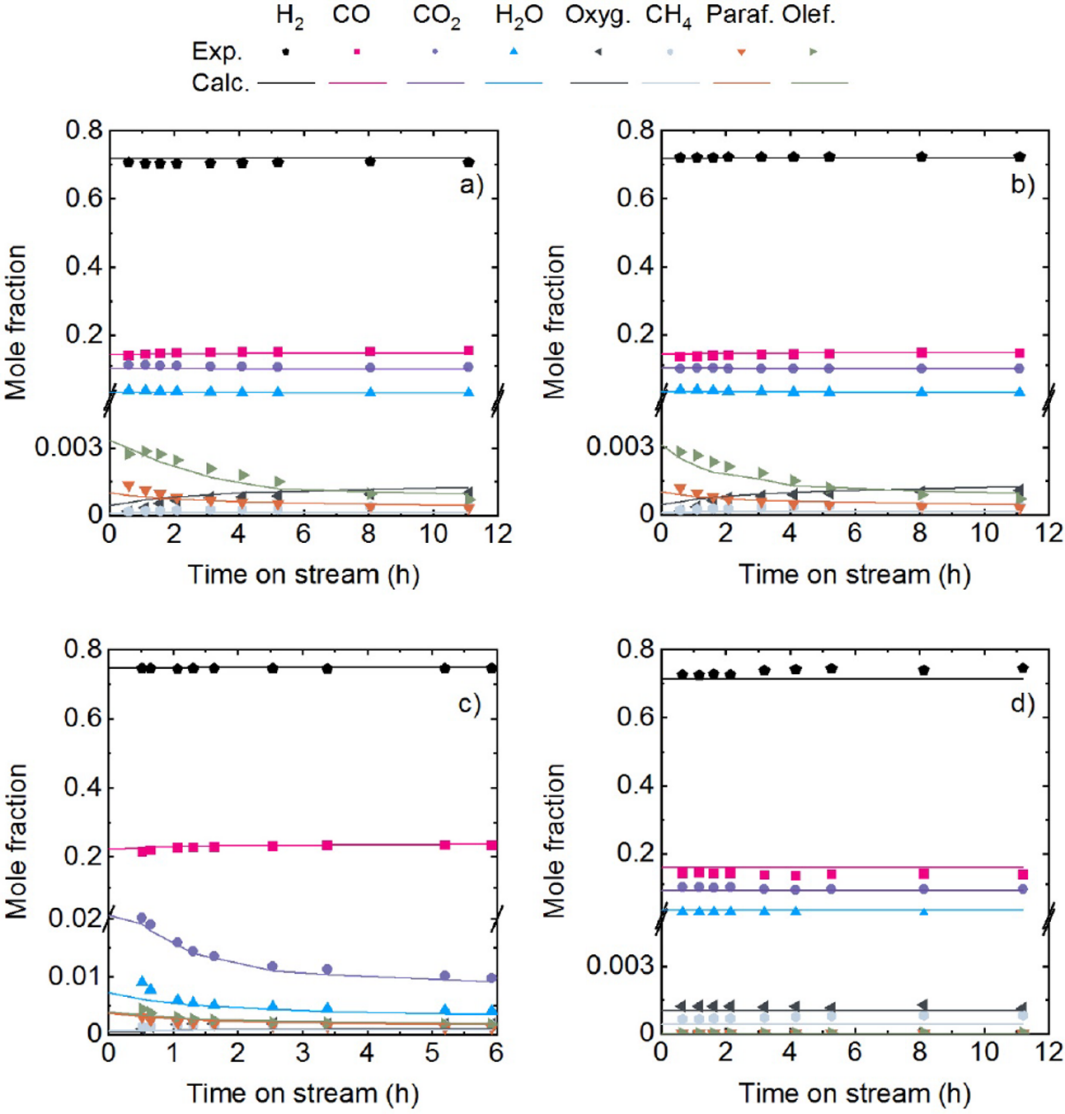
Evolution of product molar fractions with time on stream for the experimental data and calculated values. Reaction conditions: 400 °C; 30 bar; H_2_/CO_*x*_ ratio in the feed, 3; CO_2_/CO_*x*_ ratio in the feed, 0.5; space time, 5 g_cat_ h mol_C_^–1^; In_2_O_3_–ZrO_2_/SAPO-34, 2/1 (a). 375 °C; 30 bar; H_2_/CO_*x*_ ratio in the feed, 3; CO_2_/CO_*x*_ ratio in the feed, 0.5; space time, 5 g_cat_ h mol_C_^–1^; and In_2_O_3_–ZrO_2_/SAPO-34, 2/1 (b). 400 °C; 50 bar; H_2_/CO_*x*_ ratio in the feed, 3; CO_2_/CO_*x*_ ratio in the feed, 0.5; space time, 6.67 g_cat_ h mol_C_^–1^; and In_2_O_3_–ZrO_2_/SAPO-34, 2/2 (c). 400 °C; 30 bar; H_2_/CO_*x*_ ratio in the feed, 3; CO_2_/CO_*x*_ ratio in the feed, 0.5; space time, 3.33 g_cat_ h mol_C_^–1^; and In_2_O_3_–ZrO_2_/SAPO-34, 2/0 (d).

To evaluate the adequacy of fitting of the main lumps’ evolution over time on stream, Figure 2 shows the molar fractions at the reactor outlet stream, comparing calculated values (lines) with experimental data (points). Challenging conditions with high catalyst deactivation were selected as examples. Figure 2a,b differ in reaction temperature of 400 and 375 °C, respectively. Figure 2c represents a different configuration of the tandem catalyst (In_2_O_3_–ZrO_2_/SAPO–34 mass ratio of 1/1 instead of 2/1 in previous cases) and a higher pressure (50 bar instead of 30 bar). Figure 2d differs in the catalyst (no presence of SAPO–34) and space time (lower) compared with the conditions in Figure 2a. The comparison between calculated and experimental results demonstrates the model’s ability to predict the performance under different conditions. Additionally, the model satisfactorily predicts the individual behavior of each catalyst in the tandem system and the impact of the presence of an additional SAPO-34 catalyst. It is observed in Figure 2a–c, that the presence of SAPO-34 is necessary for olefin formation, but rapid deactivation leads to significant composition changes over time on stream. Both Figure 2a,b (with little difference but slightly higher olefin yield at zero time on stream at 400 °C) show a decay in olefin and paraffin concentrations over time, whereas the concentrations of oxygenates (methanol) and CH_4_ (very low concentration) increase. The deactivation kinetic model for SAPO-34 (with a deactivation order d_SAPO-34_ of 2.6) predicts, in these figures, a tendency toward constant values over time on stream for the concentrations of olefins, paraffins, and oxygenates.

As mentioned before, the model predicts the complex effects of the catalyst configuration. Figure 2c illustrates the results for a tandem catalyst with a higher SAPO-34 content (In_2_O_3_–ZrO_2_ to SAPO-34 ratio of 2/2). For this configuration, the model predicts two phenomena that have been experimentally observed: an increase in the formation of paraffins from olefins and a decrease, over time on stream, in the low concentration of CO_2_ at the reactor outlet. Figure 2d, which corresponds to the results obtained with the In_2_O_3_–ZrO_2_ catalyst, shows the model’s prediction without the deactivation effect of SAPO-34. The In_2_O_3_–ZrO_2_ catalyst is only active for the rWGS and methanol synthesis reactions, in which the concentration is much lower compared to that in previous cases due to the absence of synergy with the oxygenate-to-olefin conversion step.

The deactivation kinetics of the SAPO-34 catalyst are of great importance in the kinetic model. Figure S6a illustrates the evolution of the calculated activity with the longitudinal position and time on stream under specific reaction conditions. The preferential deactivation at the reactor inlet and the progressive trend for higher reactor longitudinal positions are characteristic of the MTO process,^52,55^ where coke is primarily formed through the adsorption of oxygenates as reactants on the acid sites, as described by the proposed kinetic eq (eq 27). In Figure S6b, it is observed that with the progression of deactivation, the normalized olefin yield tends to a stable value corresponding to the remnant activity of the catalyst.

Relating this result to the kinetic model, it can be observed that the decrease in activity and the increase in oxygenate (methanol) concentration, due to deactivation, have opposite effects on the evolution of normalized olefin formation rate over time on stream (eqs 16–18 in Table 1). Thus, the product of these two variables (Figure S6c) follows the same trend and explains Figure S6a.

### Reactor Simulation

**Figure 3:**
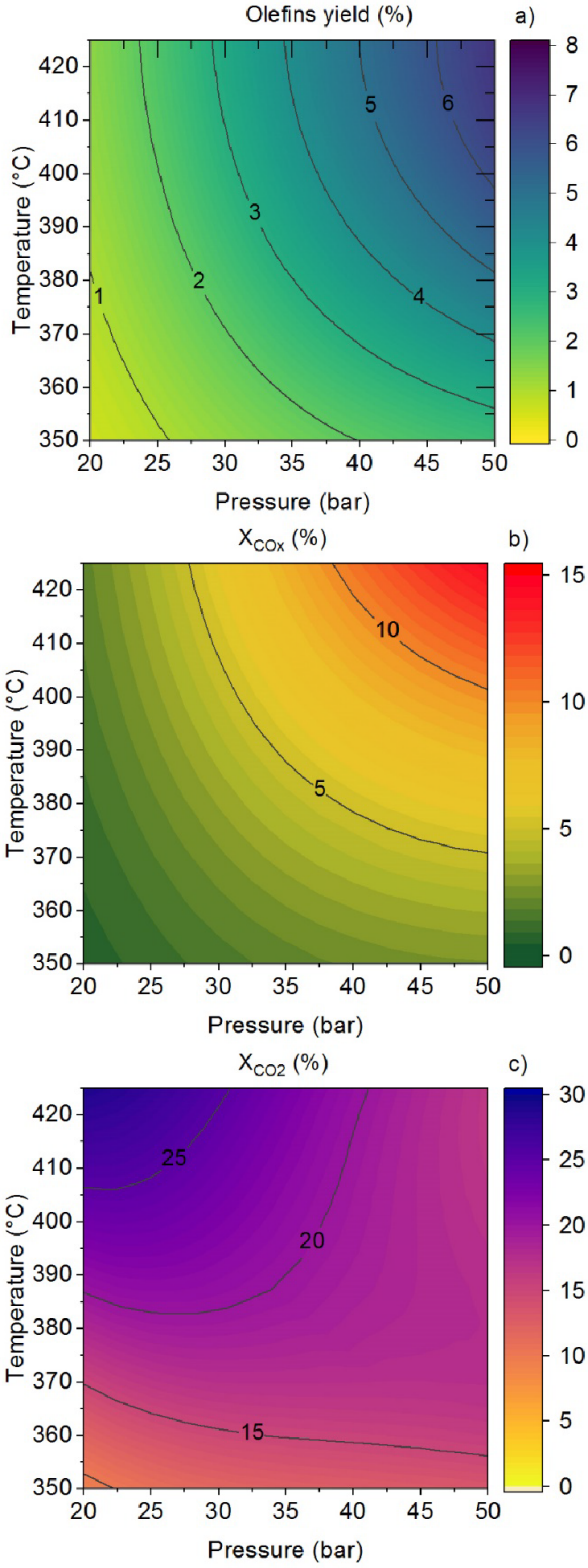
Prediction of the kinetic model on the effect of temperature and pressure on olefins yield (a), CO_*x*_ conversion (b), and CO_2_ conversion (c). Reaction conditions: H_2_/CO_*x*_ ratio in the feed, 3; CO_2_/CO ratio in the feed, 0.5; space time, 5 g_cat_ h mol_C_^–1^, and ∼0 h on stream.

The model allows for analyzing the effect of temperature, pressure, and CO_2_/CO_*x*_ ratio in the feed on the yield of light olefins, as well as on CO_2_ and CO_*x*_ conversions. As an example, the results in Figure 3 correspond to the hydrogenation of an equimolar stream of CO and CO_2_ using a catalyst with an In_2_O_3_–ZrO_2_/SAPO-34 ratio of 2/1 under specific reaction conditions and at zero time on stream (fresh catalyst). Under such conditions, the maximum olefin yield exceeds 6% (Figure 3a) with a selectivity above 60%, CO_*x*_ conversion reaches 12% (Figure 3b) and CO_2_ conversion accounts for 16% (Figure 3c) at 415 °C and 50 bar.

**Figure 4:**
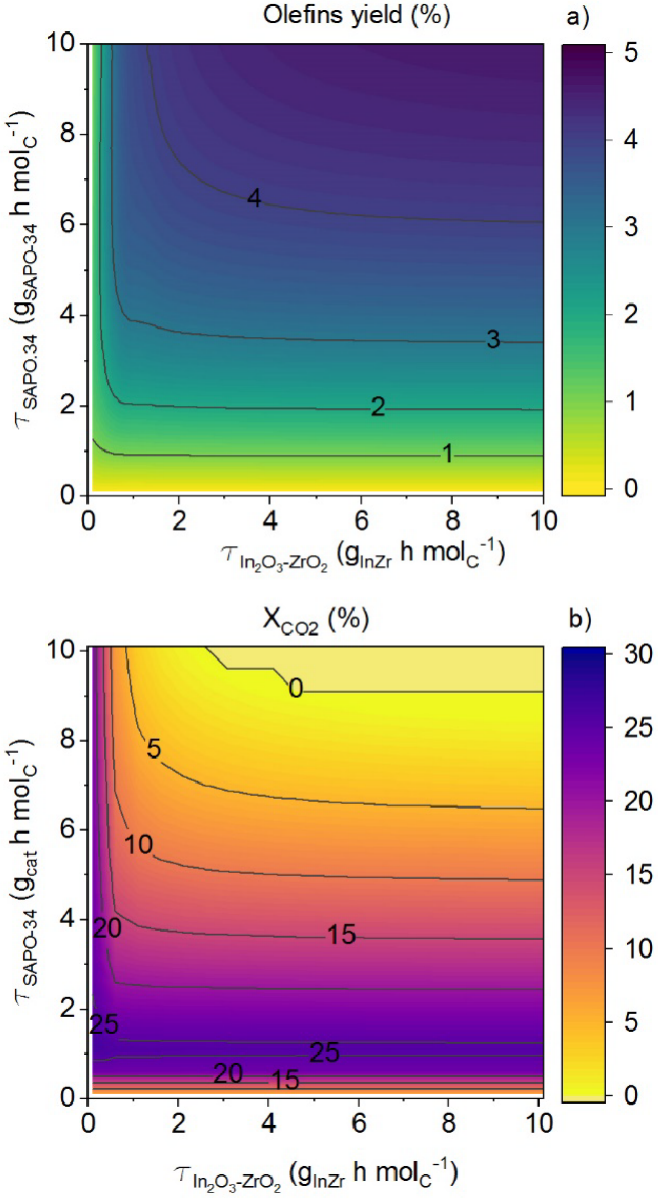
Prediction of the kinetic model on the effect of In_2_O_3_–ZrO_2_ and SAPO-34 loadings on olefins yield (a) and CO_2_ conversion (b). Reaction conditions: H_2_/CO_*x*_ ratio in the feed, 3; CO_2_/CO ratio in the feed, 0.5; 400 °C; ∼ 0 h on stream; 50 bar (a) and 30 bar (b).

It is worth noting that the methodology used in the kinetic modeling, considering the activity and deactivation independently for each of the individual catalysts, allows for predicting the performance of the tandem catalyst at different catalyst loadings. Figure 4a shows that the olefin yield increases with higher space time values of the SAPO-34 catalyst. Moreover, the In_2_O_3_–ZrO_2_/SAPO-34 ratio of 2 (corresponding to a line with slope 2) exhibits the steepest slope, indicating that it is the optimal ratio for boosting the olefin yield. Figure 4b depicts the CO_2_ conversion for different space time values of each of the individual catalysts, showing a maximum CO_2_ conversion at low SAPO-34 loadings (slightly above 1 g_SAPO-34_ h mol_C_^–1^). This circumstance predicted by the model is a direct consequence of the effect of H_2_O concentration in the medium (resulting from rWGS, methanol dehydration, and olefin formation reaction), as it hampers the rWGS reaction and leads to a complete blockage of the CO_2_ conversion.

**Figure 5:**
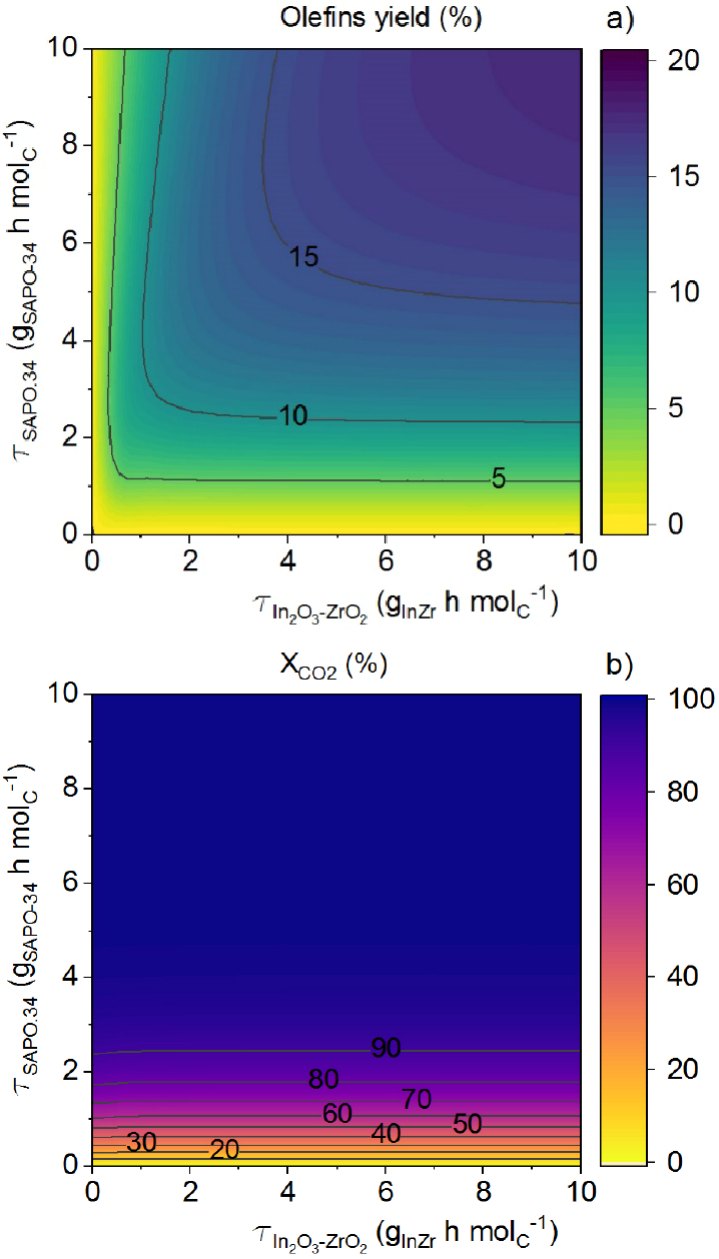
Prediction of the kinetic model on the effect of In_2_O_3_–ZrO_2_ and SAPO-34 loadings on olefins yield (a) and CO_2_ conversion (b) assuming complete and immediate H_2_O removal. Reaction conditions: H_2_/CO_*x*_ ratio in the feed, 3; CO_2_/CO ratio in the feed, 0.5; 400 °C; 30 bar; and ∼0 h on stream.

Based on the aforementioned results, aside from the significant benefit in terms of catalyst stability, the concentration of H_2_O in the reaction medium is the main limiting factor for achieving high olefin yields when the SAPO-34 content in the tandem catalyst is high. As evidence of this, a simulation was conducted for an ideal system in which H_2_O is immediately adsorbed. As shown in Figure 5a, the olefin yield surges to 17%, and complete CO_2_ conversion is achieved (Figure 5b). Therefore, two main strategies are proposed for further study seeking industrial operating conditions that imply H_2_O removal: a hydrophilic membrane reactor or *in situ* H_2_O adsorption. The latter alternative would require more investment in equipment to ensure continuous operation, that is, parallel reactors connected to an adsorption–desorption system. It is worth mentioning that significant technological advancements have been made in other catalytic processes involving CO_2_ valorization in the presence of H_2_O, such as the direct DME synthesis, which employs hydrophilic membranes^56−58^ or adsorption systems.^59^

## Conclusions

The proposed kinetic model is based on a reaction network with 12 individual steps and adequately describes the performance of an In_2_O_3_–ZrO_2_/SAPO-34 tandem catalyst in the direct production of light olefins through the hydrogenation of CO_2_/CO mixtures. The model predicts the performance in terms of product distribution, CO_2_ and CO_*x*_ conversions, and their evolution over time on stream within the 350–425 °C, 20–50 bar ranges, and a CO_2_/CO_*x*_ ratio between 0 and 1. Considering the activity of each individual catalyst in the kinetic model allows the prediction of the performance of the tandem catalyst when prepared by physical mixing with different loadings of the individual catalysts. It is also noteworthy that the deactivation of the tandem catalyst is considered through kinetic equations for the deactivation of the individual catalysts, confirming that the deactivation of SAPO-34 is the main cause and the influence of methanol as a coke precursor and that of H_2_O and H_2_ concentrations as coke formation mitigating agents.

The model proves useful for optimizing process variables and predicts an olefin yield surpassing 6% (selectivity above 60%), a CO_*x*_ conversion of 12%, and a CO_2_ conversion of 16% at 415 °C and 50 bar for a CO_2_/CO_*x*_ ratio of 0.5 in the feed. Additionally, it quantifies the effect of the concentration of methanol, H_2_O, and H_2_ on the results, suggesting the opportunity to use a hydrophilic membrane reactor. Using this reactor, the model predicts an olefin yield of 17% and complete CO_2_ conversion.

The availability of the proposed kinetic model is of significant interest for exploring the scale-up prospects of the process with catalysts utilizing In_2_O_3_–ZrO_3_ and SAPO-34 in their configuration. Moreover, the fundamental aspects described in the development of the model will be useful for kinetic modeling with other OX/ZEO catalysts under development for the direct production of olefins and other hydrocarbons from CO_2_/CO mixtures.

## Supporting Information Available

Catalysts’ properties are provided in Section S1; experimental run conditions and reproducibility in Section S2; assessment of potential mass transfer limitation in Section S3; reaction indices defined in Section S4; the methodology for kinetic data analysis described in Section S5; and, further information regarding the fitting obtained with the model and simulation in Section S6 (PDF)
