# Supplementary material for: Kinetic Model for the Direct Conversion of CO2/CO into Light Olefins over an In2O3–ZrO2/SAPO-34 Tandem Catalyst
Source: ACS Sustain Chem Eng. 2024 Jan 18;12(4):1616–24. doi: 10.1021/acssuschemeng.3c06914 (PMC10828986; doi:10.1021/acssuschemeng.3c06914)
Supplement: Supplementary file 1 — sc3c06914_si_001.pdf [file sc3c06914_si_001.pdf]

Supporting information for:

Kinetic model for the direct conversion of CO_2/CO into light olefins over an In_2O_3-ZrO_2/SAPO-34 tandem catalyst

Ander Portillo, Onintze Parra, Andres T. Aguayo, Javier Ereña, Javier Bilbao, Ainara Ateka*

Department of Chemical Engineering, University of the Basque Country UPV/EHU, P.O. Box 644, 48080 Bilbao, Spain

*Corresponding author. Tel.: +34-94-6015361. E-mail address: ainara.ateka@ehu.eus

---

Number of pages: 15

Number of Figures: 6

Number of Tables: 3

Section S1. Catalysts properties

Table S1. Chemical, physical and acidic properties of the catalysts used in tandem.

**Chemical and structural properties of In_2O_3-ZrO_2**		
	**In_2O_3**	**ZrO_2**
**XRF analysis: Metal content (%)**	67.1	32.9
**XRD analysis: Crystal size (nm)**	21	8
**Physical properties of the catalysts**		
	**In_2O_3-ZrO_2**	**SAPO-34**
**S_{BET} (m^2 g^{-1})**	86	652
**V_{pore} (cm^3 g^{-1})**	0.23	0.23
**$V_{micro\ pore}$ (cm^3 g^{-1})**	-	0.22
**dp (nm)**	9	1.5
**Acidic properties of SAPO-34**		
		**SAPO-34**
**Total acidity (μmol$_{NH3}$ g^{-1})**		652
Weak (%)		12.7
Medium (%)		20.6
Strong (%)		66.7

Section S2. Experimental run conditions

Table S2. Kinetic runs considered for the kinetic modeling. W and M correspond to H_2O and methanol, respectively.

Run #	T (ºC)	P (bar)	τ ($g_{cat}h\ mol_C^{-1}$)	M/A	TOS (h)	$H_2/CO/CO_2$	Cofeed	Bed
1	350	30	5	2	7	03/01/2000	–	Single
2	350	30	5	2	7	3/0.75/0.25	–	Single
3	350	30	5	2	7	3/0.5/0.5	–	Single
4	350	30	5	2	7	3/0/1	–	Single
5	375	30	5	2	16	3/0.5/0.5	–	Single
6	375	30	5	2	16	3/0/1	–	Single
7	375	30	5	2	14	3/0/1	–	Single
8	400	20	2	2	14	3/0.5/0.5	–	Single
9	400	20	2	2	14	3/0.5/0.5	–	Single
10	400	20	5	2	7	03/01/2000	–	Single
11	400	20	5	2	14	3/0.5/0.5	–	Single
12	400	20	5	2	15	3/0/1	–	Single
13	400	20	10	2	14	3/0.5/0.5	–	Single
14	400	30	2	0	14	3/0/1	M	Single
15	400	30	2	0	7	3/0/1	M	Single
16	400	30	3	Inf.	14	03/01/2000	–	Single
17	400	30	3	Inf.	14	03/01/2000	–	Single
18	400	30	3	Inf.	14	3/0.5/0.5	–	Single
19	400	30	4	4	7	03/01/2000	–	Single
20	400	30	5	2	16	03/01/2000	–	Single
21	400	30	5	2	70	03/01/2000	–	Single
22	400	30	5	2	16	03/01/2000	–	Single
23	400	30	5	2	16	03/01/2000	–	Single
24	400	30	5	2	16	3/0.75/0.25	–	Single
25	400	30	5	2	14	1/0.5/0.5	–	Single
26	400	30	5	2	14	2/0.5/0.5	–	Single
27	400	30	5	2	7	3/0.5/0.5	–	Single
28	400	30	5	2	16	3/0.25/0.75	–	Single
29	400	30	5	2	499	3/0/1	–	Single
30	400	30	5	2	16	3/0/1	–	Single
31	400	30	5	2	72	3/0/1	–	Single
32	400	30	5	2	7	3/0/1	M	Single
33	400	30	5	2	14	3/0/1	M	Single
34	400	30	5	2	14	3/0/1	M	Single
35	400	30	5	2	14	03/01/2000	W	Single
36	400	30	5	2	14	3/0.5/0.5	W	Single
37	400	30	5	2	15	3/0/1	W	Single
38	400	30	5	2	15	3/0/1	WM	Single

Table S2. Continuation.

Run #	T (ºC)	P (bar)	τ ($g_{cat}h\ mol_C^{-1}$)	M/A	TOS (h)	$H_2/CO/CO_2$	Cofeed	Bed
39	400	30	5	2	7	3/0.5/0.5	WM	Single
40	400	30	5	2	15	3/0/1	WM	Single
41	400	30	6	1	7	03/01/2000	–	Single
42	400	30	6	1	14	3/0.5/0.5	–	Single
43	400	30	10	0.5	14	03/01/2000	–	Single
44	400	30	10	0.5	14	03/01/2000	–	Single
45	400	30	10	0.5	14	03/01/2000	–	Single
46	400	30	10	0.5	16	3/0.5/0.5	–	Single
47	400	30	10	0.5	16	3/0/1	–	Single
48	400	30	10	0.5	14	03/01/2000	–	Dual
49	400	30	10	0.5	7	03/01/2000	–	Dual
50	400	30	10	0.5	14	3/0.5/0.5	–	Dual
51	400	30	10	0.5	14	3/0.5/0.5	–	Dual
52	400	30	10	2	14	03/01/2000	–	Single
53	400	30	23	0.2	16	03/01/2000	–	Single
54	400	40	5	2	7	03/01/2000	–	Single
55	400	40	5	2	7	03/01/2000	–	Single
56	400	40	5	2	7	3/0.75/0.25	–	Single
57	400	40	5	2	7	3/0.5/0.5	–	Single
58	400	40	5	2	14	3/0.5/0.5	–	Single
59	400	40	5	2	14	3/0/1	–	Single
60	400	40	5	2	14	3/0/1	–	Single
61	400	40	6	1	7	03/01/2000	–	Single
62	400	50	5	2	7	03/01/2000	–	Single
63	400	50	5	2	7	3/0.5/0.5	–	Single
64	400	50	5	2	7	3/0.5/0.5	–	Single
65	400	50	5	2	14	3/0/1	–	Single
66	400	50	5	2	14	3/0/1	–	Single
67	400	50	10	0.5	14	3/0.5/0.5	–	Single
68	400	30	10	0.5	7	03/01/2000	–	Dual
69	425	30	5	2	16	03/01/2000	–	Single
70	425	30	5	2	16	3/0/1	–	Single

*τ ($g_{cat}h\ mol_C^{-1}$): defined as the catalyst loading per ($CO+CO_2$) mol flowrate in the feed.

15 repeated conditions (11 repeated twice, 2 repeated 3 times, and 1 repeated 4 times).

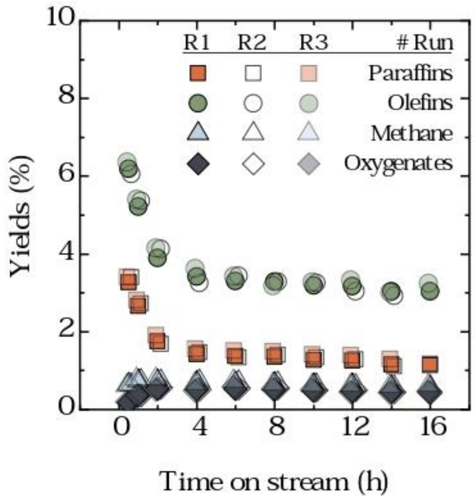

Figure S1. Experimental run reproducibility for three repeated experiments at the following conditions: a) 400 ºC; 30 bar; CO_2/CO_x in the feed, 1; space time, 5 g_{cat}·h mol_C^{-1}.

Section S3. Assessment of potential mass transfer limitation

The compliance with the Weisz-Prater criterion (eq (S1) was carried out to ascertain the absence of mass transfer constraints within the catalyst particles. The calculation was performed following the methodology suggested by García-Sánchez et al.[1] and confirmed the absence of diffusional limitations to the flow of reactants within the porous structure of the catalyst. The calculated Weisz-Prater modulus (C_{WP}) for the fastest methanol formation reaction (from CO) in the In_2O_3-ZrO_2 catalyst accounts for 0.015. This value is in accordance with the values in the literature for Cu-Zn-Al catalysts ($C_{WP} < 0.03$, for Leonzio et al.[2]).

$$C_{WP} = \frac{(-r)R^2}{D_e C_{reactant}} < 1 \quad \text{(S1)}$$

where (-r) is the reaction rate, R is the catalyst particle radius, D_e is the effective diffusivity, $C_{reactant}$ is the concentration of the reactant. The properties of the catalysts (to calculate D_e) are: porosity, ε= 0.23; tortuosity, τ= 1.17; Knudsen diffusivity, D_a=5 10^{-9} m^2s^{-1}.

Moreover, the absence of mass transfer limitations has also been experimentally proven as observed in Figures S2 (with runs carried out with different particle size, but same size for both the In_2O_3-ZrO_2 and SAPO-34 catalysts) and S3 (with runs carried out varying the total flow rate at the reactor inlet). Figure S2 ascertains the absence of mass transfer constraints within the studied particle range. This indicates that the particle sizes employed in the kinetic modeling are appropriate (in the 125-250 µm range for the In_2O_3-ZrO_2 catalyst and 300-400 µm range for the SAPO-34 catalyst. The distinct sizes for each catalyst were chosen to facilitate post-reaction separation and enable individual analysis of the respective catalysts). The results presented in Figure S3 highlight the absence of external diffusion constraints for the total gas flow rate used in the kinetic modeling (60 cm^3 min^{-1}).

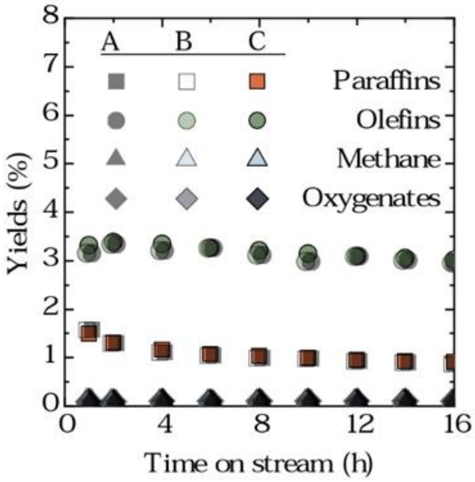

Figure S2. Evolution of products yield over time on stream for different particle size catalysts. Reaction conditions: 400 ºC; 30 bar; CO_2/CO_x ratio in the feed, 0.5; and space time 5 g_{cat} h mol_C^{-1}. Particle sizes: A, 60-100 µm; B, 125-400 µm and C, 600-900 µm.

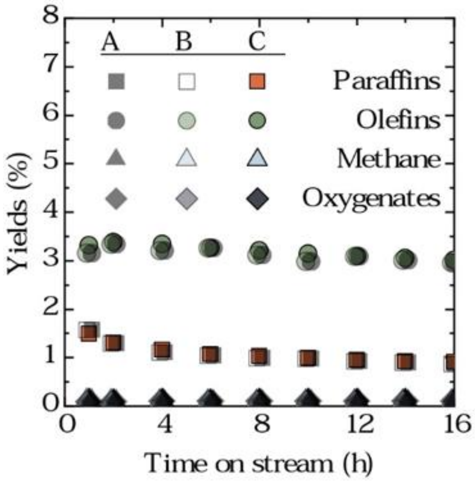

Figure S3. Evolution of products yield over time on stream for different particle size catalysts. Reaction conditions: 400 ºC; 30 bar; CO_2/CO_x ratio in the feed, 0.5; and space time 5 g_{cat} h mol_C^{-1}. Flowrate is referred in cm^3 min^{-1}.

Section S4. Reaction indices

The results were quantified by calculating olefin yields, Y_i (eq S2), CO_2 conversion, X_{CO2} (eq S3), and CO_x conversion, X_{COx} (eq S4) based on the molar flows of the reactants and products in C content units as:

$$Y_i = \frac{n_i F_i}{F^0_{CO_x}} 100 \qquad \text{(S2)}$$

where n_i is the number of carbon atoms of each i product, F_i the molar flow rate of the i product at the outlet and $F^0_{CO_x}$ the total molar flow rate of CO_x ($CO+CO_2$) in the feed.

$$X_{CO_2} = \frac{F^0_{CO_2} - F_{CO_2}}{F^0_{CO_2}} 100 \qquad \text{(S3)}$$

where $F^0_{CO_2}$ and F_{CO_2} are the molar flow rate of CO_2 at the inlet and outlet of the reactor, respectively.

$$X_{CO_x} = \frac{F^0_{CO_x} - F_{CO_x}}{F^0_{CO_x}} 100 \qquad \text{(S4)}$$

where $F_{CO_x}$is the molar flow of ($CO + CO_2$) at the outlet of the reactor.

Section S5. Methodology for kinetic data analysis

The mathematical approach used for analyzing the kinetic data in this study has been described in detail in other works by the authors on the kinetic modeling of catalytic processes with complex reaction schemes subject to coke deactivation.[3–5] In summary, the calculation of the apparent kinetic constants involves fitting the results of integrating the mass conservation equations (convection-dispersion-reaction equations) for each compound (or lump) in the reaction network (eq S5) to the corresponding experimental data of concentration evolution with time on stream.

$$\varepsilon \frac{\partial \mathbf{y_i}}{\partial t} = -\frac{\partial}{\partial z}\left(\upsilon \mathbf{y_i} - D\frac{\partial \mathbf{y_i}}{\partial z}\right) + \frac{RT}{PN_C}\rho \mathbf{r_i} \qquad i=1,\ldots,n_l \qquad \text{(S5)}$$

In this equation, a vector of each lumps' molar fraction $\mathbf{y_i}$ (z,t) is considered. The equation is defined for a time value t>0 and a longitudinal position in the reactor 0<z<L (catalytic bed length). ε is the effective bed-particle porosity, υ is the linear velocity, D is the effective dispersion coefficient of the gas (not relevant in this case), R the universal gas constant, T is the temperature, P the total pressure, N_C is the ratio between the (carbon molar flow rate)/(total molar flow rate), ρ is the density of the catalytic bed, $\mathbf{r_i}$ vector comprises the formation rates of each i lump and n_l the number of components.

The system of parabolic partial differential equations was solved by transforming it into a system of ordinary differential equations.[6] This system was integrated using an implicit Runge-Kutta method, and the kinetic parameters were calculated by minimizing the objective function vector (**OF**) defined in eq S6, where the first term is related to the kinetics of each j reaction step in the reaction network at t=0, and the second term is related to the deactivation kinetics. Both terms are defined as the differences between the experimental results (molar fractions at t=0 and at the given t time on stream, that is, $y_{i,0}$

and y_i, respectively) and the corresponding values calculated with the kinetic model. A modification of the Levenberg-Marquardt algorithm[7,8] was used for computation.

$$\mathbf{OF} = \begin{pmatrix} \sum_{i=1}^{n_l} \omega_i \sum_{n=1}^{n_{e,0}} \frac{\xi}{n_{e,0}} \left(y_{i,0}^e - y_{i,0}\right)_n^2 \\ \sum_{i=1}^{n_l} \omega_i \sum_{n=1}^{n_{e,d}} \frac{\xi}{n_{e,d}} \left(y_i^e - y_i\right)_n^2 \end{pmatrix} \tag{S6}$$

where ω_i represents the weight factor assigned to each component i, ξ is the number of repeated runs under the same reaction conditions, and $n_{e,0}$ and $n_{e,d}$ denote the total number of experimental data used for calculating the first and second terms of the objective function (**OF**), respectively.

The weight factors (gathered in Table S3) are calculated as the inverse of the variance of each component according to the expression defined by Constantinides and Mostoufi[9] (eq (S7)):

$$\omega_i = \frac{1/\sigma_i^2}{\left[\frac{1}{\sum_{m=1}^{nv} n_e}\right]\left[\sum_{m=1}^{nv} \sum_{n=1}^{ne} \left(1/\sigma_j^2\right)\right]} \tag{S7}$$

where σ_i^2 and σ_j^2 are the variances for each distribution of experimental results of component *i* at each *j* reaction stage.

In the absence of repeated runs, no initial variances can be estimated and so, the weight factor is established to be inversely proportional to the average concentration of each component in the studied condition range (eq (S8)):

$$\omega_i = \frac{1}{\sum_{j=1}^{n_{exp}} x_i} \tag{S8}$$

Table S3. Weigh factor of the components.

H_2	CO	CO_2	H_2O	Oxygenates	CH_4	Paraffins	Olefins
$1.42\ 10^0$	$6.12\ 10^0$	$1.01\ 10^1$	$3.64\ 10^1$	$1.38\ 10^3$	$9.43\ 10^2$	$7.68\ 10^2$	$5.46\ 10^2$

Additionally, a statistic study based on the analysis of variances was carried out to assess the significance of the model. The method used is based on the Fisher test, and compares the experimental $F_{a\text{-}e}$ value with the critical value of Fisher distribution, $F_{1-\alpha(v_a,v_e)}$, as shown in eq (S9). The experimental $F_{a\text{-}e}$ value is calculated as the ratio between the variance of the lack of fit of the model (s_a^2) and that of the experimental error calculated from repeated runs (s_e^2). Each variance is calculated as the ratio between the sum of square errors and the corresponding degree of freedom. That is, SSE_a and v_a for the lack of fit and; SSE_e and v_e for the experimental error. Note that $F_{1-\alpha(v_a,v_e)}$ is the critical value of Fisher distribution for a 95% confidence interval (α=0.05). When the experimental $F_{a\text{-}e}$ is lower than the critical value $F_{1-\alpha(v_a,v_e)}$ the lack of fit is not significant, and therefore, the model is suitable to predict the evolution of the experimental data.[3,10,11]

$$F_{a-e} = \frac{\frac{SSE_a}{v_a}}{\frac{SSE_e}{v_e}} = \frac{s_a^2}{s_e^2} < F_{1-\alpha(v_a,v_e)} \qquad \text{(S9)}$$

Section S6. Fitting and simulation

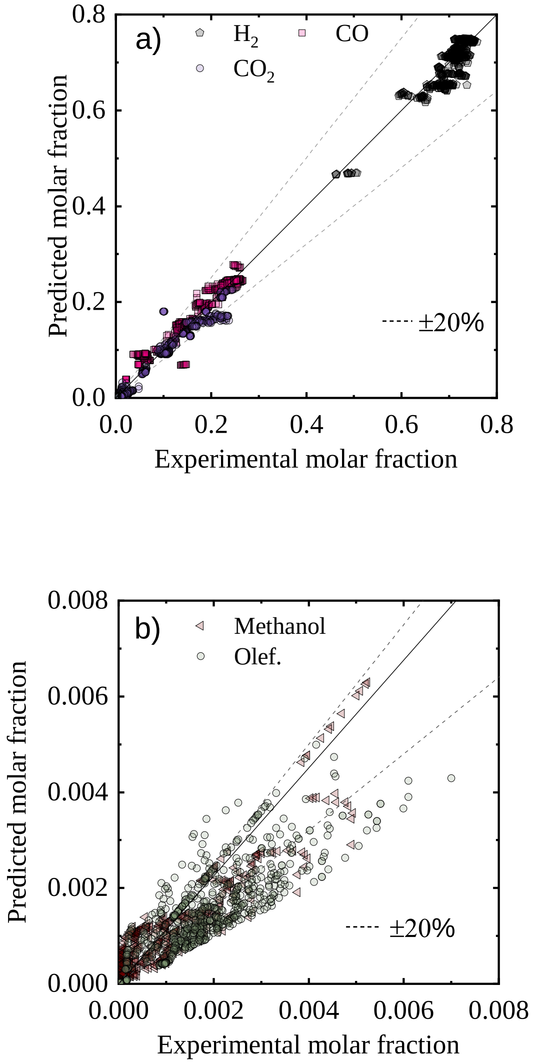

Figure S4. Detailed comparison of the calculated values of the molar fractions with experimental results for the reactants (a) and methanol and olefins (b). The solid and dashed lines represent the diagonal and the ±20% error lines, respectively.

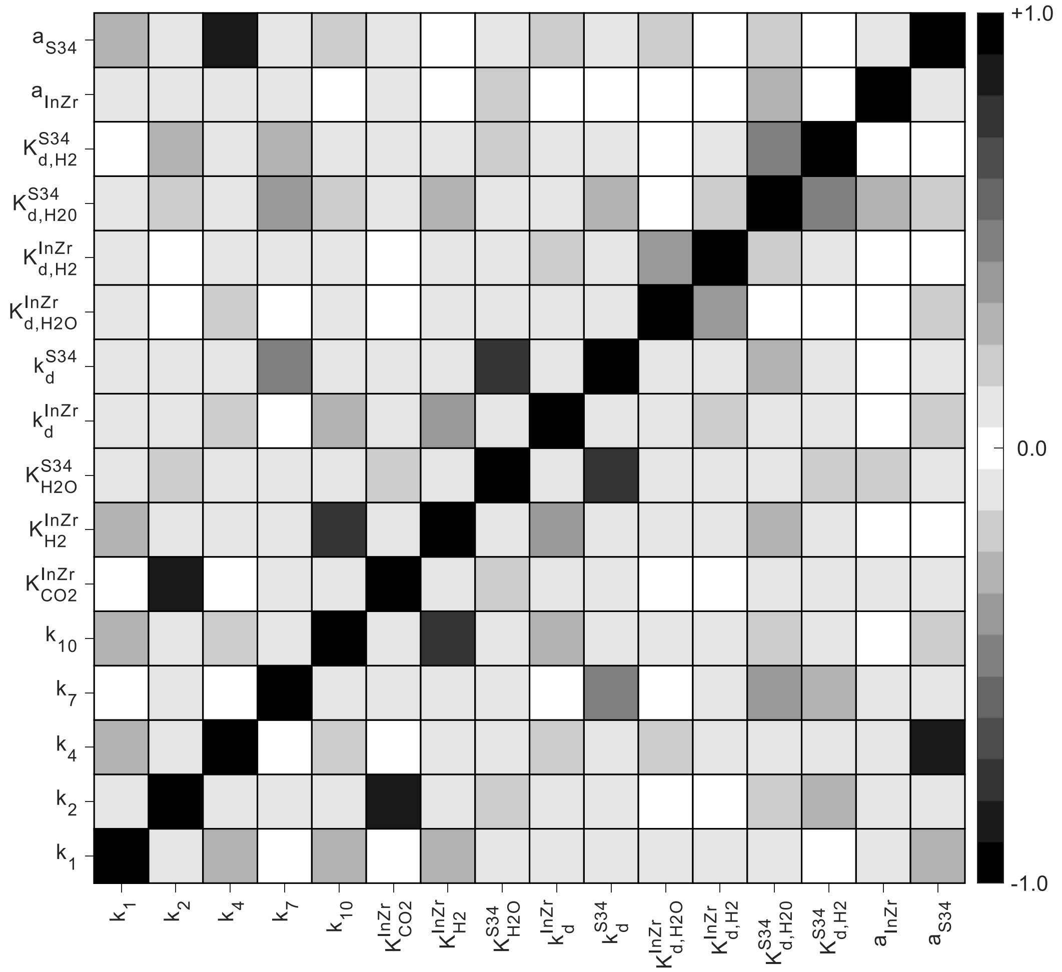

Figure S5. Covariance matrix.

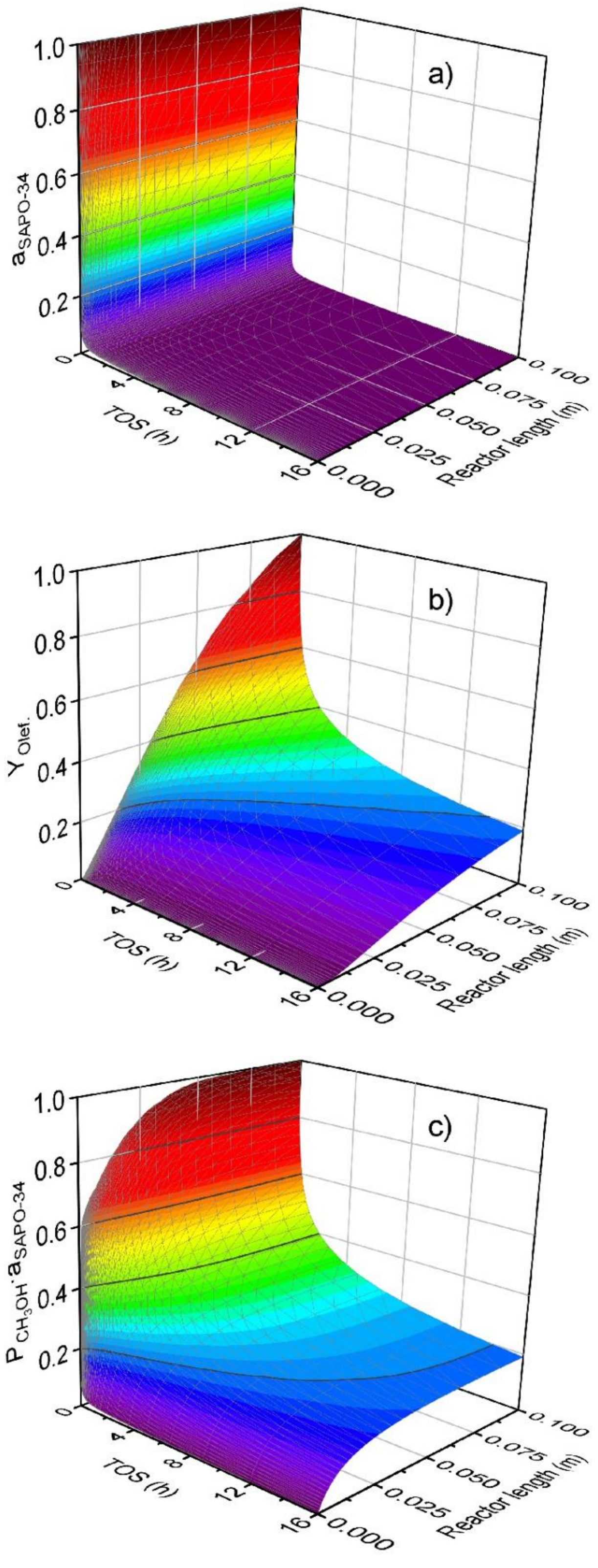

Figure S6. Evolution with time on stream and reactor length of activity (a), olefins normalized yield (b) and product of methanol concentration and activity (c).

REFERENCES

(1) García-Sánchez, J. T.; Valderrama-Zapata, R.; Acevedo-Córdoba, L. F.; Pérez-Martínez, D.; Rincón-Ortiz, S.; Baldovino-Medrano, V. G. Calculation of Mass Transfer Limitations for a Gas-Phase Reaction in an Isothermal Fixed Bed Reactor: Tutorial and Sensitivity Analysis. *ACS Catal.* **2023**, *13*, 6905–6918. https://doi.org/10.1021/acscatal.3c01282.

(2) Leonzio, G. Mathematical Modeling of a Methanol Reactor by Using Different Kinetic Models. *J. Ind. Eng. Chem.* **2020**, 85, 130-140. https://doi.org/10.1016/j.jiec.2020.01.033.

(3) Cordero-Lanzac, T.; Aguayo, A. T.; Gayubo, A. G.; Castaño, P.; Bilbao, J. Simultaneous Modeling of the Kinetics for n-Pentane Cracking and the Deactivation of a HZSM-5 Based Catalyst. *Chem. Eng. J.* **2018**, *331*, 818–830. https://doi.org/10.1016/J.CEJ.2017.08.106.

(4) Ateka, A.; Portillo, A.; Sánchez-Contador, M.; Bilbao, J.; Aguayo, A. T. Macro-Kinetic Model for CuO–ZnO–ZrO_2@SAPO-11 Core-Shell Catalyst in the Direct Synthesis of DME from CO/CO_2. *Renew. Energy* **2021**, *169*, 1242-1251. https://doi.org/10.1016/j.renene.2021.01.062.

(5) Cordero-Lanzac, T.; Hita, I.; García-Mateos, F. J.; Castaño, P.; Rodríguez-Mirasol, J.; Cordero, T.; Bilbao, J. Adaptable Kinetic Model for the Transient and Pseudo-Steady States in the Hydrodeoxygenation of Raw Bio-Oil. *Chem. Eng. J.* **2020**, *400*, 124679. https://doi.org/10.1016/j.cej.2020.124679.

(6) Skeel, R. D. Global Error Estimation and the Backward Differentiation Formulas. *Appl. Math. Comput.* **1989**, *31*, 197–208. https://doi.org/https://doi.org/10.1016/0096-3003(89)90119-7.

(7) Levenberg, K. A Method for the Solution of Certain Problems in Least Suares. *Q. Appl. Math.* **1944**, *2*, 164–168.

(8) Marquardt, D. W. An Algorithm for Least-Squares Estimation of Nonlinear Parameters. *J. Soc. Ind. Appl. Math.* **1963**, *11*, 431–441. https://doi.org/10.1137/0111030.

(9) Constantinides, A.; Mostoufi, N. *Numerical Methods for Chemical Engineers with MATLAB Applications*; Prentice Hall PTR: Upper Saddle River, NJUnited States, 1999.

(10) Pérez-Uriarte, P.; Ateka, A.; Gayubo, A. G.; Cordero-Lanzac, T.; Aguayo, A. T.; Bilbao, J. Deactivation Kinetics for the Conversion of Dimethyl Ether to Olefins over a HZSM-5 Zeolite Catalyst. *Chem. Eng. J.* **2017**, *311*, 367–377. https://doi.org/10.1016/J.CEJ.2016.11.104.

(11) Roohollahi, G.; Kazemeini, M.; Mohammadrezaee, A.; Golhosseini, R. Chemical Kinetic Modeling of I-Butane and n-Butane Catalytic Cracking Reactions over HZSM-5 Zeolite. *AIChE J.* **2012**, *58*, 2456–2465. https://doi.org/https://doi.org/10.1002/aic.12750.
